# Reproducibility and Consistency of Isolation Protocols for Fibroblasts, Smooth Muscle Cells, and Epithelial Cells from the Human Vagina

**DOI:** 10.3390/cells14020076

**Published:** 2025-01-08

**Authors:** Jayson Sueters, Rogier Schipperheijn, Judith Huirne, Theo Smit, Zeliha Guler

**Affiliations:** 1 Department of Gynaecology, Amsterdam UMC—Location VUmc, De Boelelaan 1117, 1105 AZ Amsterdam, The Netherlands; j.sueters@amsterdamumc.nl (J.S.); j.huirne@amsterdamumc.nl (J.H.); th.smit@amsterdamumc.nl (T.S.); 2 Reproductive Biology Laboratory, Amsterdam UMC—Location AMC, Meibergdreef 9, 1105 AZ Amsterdam, The Netherlands; 3 Amsterdam Reproduction and Development Research Institute, Meibergdreef 9, 1105 AZ Amsterdam, The Netherlands; 4 Amsterdam UMC—Location UvA, Faculty Medicine, University of Amsterdam, Meibergdreef 15, 1105 AZ Amsterdam, The Netherlands; 5 Department of Medical Biology, Amsterdam UMC—Location AMC, Meibergdreef 9, 1105 AZ Amsterdam, The Netherlands; 6 Department of Obstetrics and Gynecology, Amsterdam UMC—Location AMC, Meibergdreef 9, 1105 AZ Amsterdam, The Netherlands

**Keywords:** cell isolation, vaginal epithelial cells, fibroblasts, smooth muscle cells, tissue engineering, vagina reconstruction

## Abstract

(1) Background: For the reconstruction of a human vagina, various surgical procedures are available that are often associated with complications due to their failure to mimic the physiology of the human vagina. We recently developed a vascularized, organ-specific matrix from healthy human vaginal wall tissue with suitable biomechanical properties. A superior graft would require further extensive colonization with autologous vaginal cells to reduce complications upon implantation. However, reports on isolation of vaginal cells from biopsies are scarce, and published protocols rarely contain sufficient details. In this study, we aimed to examine protocols for inconsistencies and identify (where possible) the optimal protocol in terms of reproducibility and efficiency for isolation of human vaginal fibroblasts (FBs), epithelial cells (VECs), and smooth muscle cells (SMCs). Overall, this study aims to guide other researchers and aid future tissue engineering solutions that rely on autologous cells. (2) Methods: A total of 41 isolation protocols were tested: four protocols specific to FBs, 13 protocols for VECs, and 24 protocols for SMCs. Protocols were derived from published reports on cell isolation by enzymes, with exclusion criteria including the need for specialized equipment, surgical separation of tissue layers, or missing protocol details. Enzymatic digestion with collagenase-I, collagenase-IV, and dispase-II was used for isolation of VECs, collagenase-IV for isolation of SMCs, and collagenase-IA for isolation of FBs. Fluorescent immunostaining was applied to identify VECs with cytokeratin, SMCs with desmin, endothelial cells with UEA-1, and FBs with vimentin. Protocols were assessed based on (>95%) homogeneity, duplicate consistency, cell viability, and time to first passage. (3) Results: A total of 9 out of the 41 protocols resulted in isolation and expansion of vaginal FBs. This involved 1 out of 13 VEC protocols, 6 out of 24 SMC protocols, and 2 out of 2 FB protocols. Isolation of vaginal SMCs or VECs was not achieved. The best results were obtained after digestion with 0.1% collagenase-IV, where pure FB colonies formed with high cell viability. (4) Conclusions: Today, vaginoplasty is considered the gold standard for surgically creating a neovagina, despite its considerable drawbacks and limitations. Tissue-engineered solutions carry great potential as an alternative, but cell seeding is desired to prevent complications upon implantation of grafts. In this study, we examined isolation of human vaginal FBs, SMCs, and VECs, and identified the most efficient and reliable protocol for FBs. We further identified inconsistencies and irreproducible methods for isolation of VECs and SMCs. These findings aid the clinical translation of cell-based tissue engineering for the reconstruction and support of vaginas, fulfilling unmet medic needs.

## 1. Introduction

Partial or complete absence of a healthy, functional vagina can be caused by various medical disorders (Mayer–Rokitansky–Küster–Hauser syndrome, cloacal malformations, endocrine abnormalities, gender incongruency, and disorder of sex development) or acquired injuries (cancer, radiation, trauma, and genital mutilation), and hampers the individual’s quality of life and psychological well-being [1]. Many solutions are available for the creation and repair of a vagina, but none is without limitations, risks, or drawbacks. Non-surgical treatment is not always optional and imposes mental agony and distress on the patient, as the wall of the vaginal dimple needs to be forcefully stretched daily; this is not always successful and regularly requires surgery as a secondary treatment [2]. Current surgery techniques (vaginoplasty) rely on dissection of a neovaginal cavity with subsequent graft lining. Typical (autologous) neovaginal grafts are derived from the skin, intestine, or peritoneum. They are associated with donor-site morbidities, stenosis, and graft contracture, as they insufficiently mimic the physiological properties of the human vaginal wall [3,4,5,6,7,8,9,10]. Meanwhile, donor grafts pose risks of immunorejection [11] and the transmission of infections and diseases [12,13]. In the last decennia, tissue-engineered solutions have demonstrated their potential in clinical applications. An ideal tissue-engineered replacement is biocompatible and resembles the native tissue in structure and function. Therefore, our group has previously worked on the chemical removal of donor cells from human vaginal wall tissue, to develop a decellularized vaginal matrix (DVM) with preserved tissue structure and composition and without risk of immune rejection. We demonstrated that the structural integrity, important extracellular matrix (ECM) proteins, and the vascular network were preserved upon decellularization, and the donor deoxyribonucleic acid (DNA) quantity was reduced below thresholds that could induce immune responses upon implantation. However, decellularization compromised the biomechanical properties (strain, stress, and elastic modulus). Therefore, the use of our DVM as a neovaginal graft requires further functionalization to restore its biomechanical properties. This can be achieved by in vitro colonization with host-derived cells, but requires isolation of the various primary autologous cell types that are naturally present in the vagina. For transgender individuals, this could be autologous cells from e.g., penile skin, the urethra, or buccal mucosa. Moreover, autologous cell seeding is not only crucial for full neovaginal reconstruction but also aids clinical applications of tissue-engineered constructs for gynecological disorders that require support of the vaginal wall, such as stress urinary incontinence (SUI) and pelvic organ prolapse (POP). It has been hypothesized that pre-seeding vaginal implants can reduce the clinical complications seen in SUI or POP treatment [14,15,16,17,18]. Biopsy-obtained autologous vaginal cells have even been developed into completely cell-based neovaginal constructs without the need for a biomaterial scaffold [19,20]. For example, in vitro three-dimensional (3D) self-assembly of autologous vaginal epithelial and stromal cells without scaffolding material can be used to create a vagina that resembles the native vagina in function and biological composition [19].

The human vaginal wall consists of a layer of epithelium, lamina propria, muscularis, and adventitia [21,22]. The superficial epithelium is composed of vaginal epithelial cells (VECs). The adjacent lamina propria contains fibroblasts (FBs), collagen, elastic fibers, and thin-walled blood vessels [21,22]. The muscularis is formed from smooth muscle cells (SMCs), and the outer adventitia layer contains connective tissue with blood vessels and nerves [21,22]. Application of tissue-engineered grafts may necessitate the isolation and utilization of one or more of these cell types that are native to the vagina.

Methods to retrieve primary cells are based on mechanical isolation, enzymatic digestion, magnetic isolation, or density gradient separation. The preferred method relies on the specific cell type but also on a broad range of surrounding factors (such as pH, medium constituents, environmental temperature, oxygen concentration, cell plate stiffness, movement, etc.). Publications widely report isolation of various cell types from human organs and tissues, including peripheral blood [23], foreskin [24,25], skin [26,27,28,29,30], and blood vessels [31,32,33], among others. Yet, protocols for isolation of primary vaginal cells are scarce, and have often either been optimized for retrieval from and application in animals, rather than by clinical trials [34,35], or lack essential details (Table 1), such as enzyme concentrations, digestion period, the procedure for cell pellet retrieval, and examination of cell viability. Moreover, the academic community is generally focused on reports with successful outcomes. For instance, not all journals are willing to publish methods or procedures with ‘failed’ outcomes. Researchers are inclined to redo experiments in case of unexpected outcomes rather than consider a theory to be contradicted, and publications generally contain only the best obtained results while lacking information on their success ratio. By also reporting inconsistencies or unsuccessful methods, we can help fellow scientists to avoid spending precious time, personnel, and resources on theories or protocols that are doomed to fail from the beginning.

**Figure 1:**
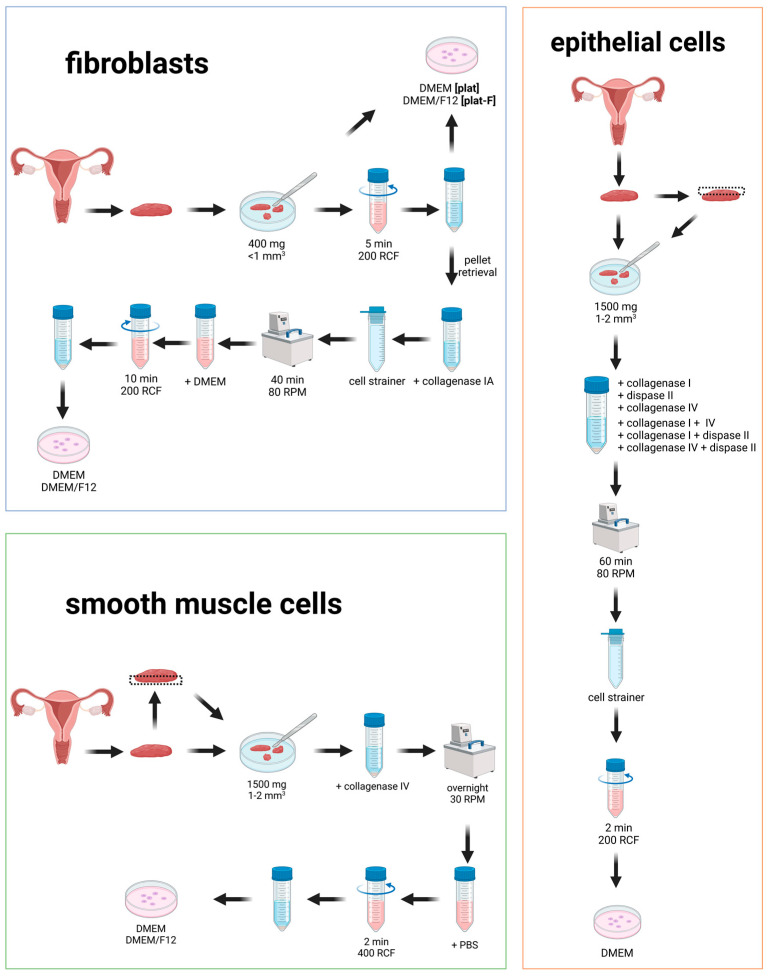
Isolation protocols were tested for fibroblasts (blue), smooth muscle cells (green), and epithelial cells (orange) from human vaginal wall tissue. This illustration provides a schematic overview of the tested protocols.

The aim of this study is to identify protocols for the efficient isolation of the three major cell types that constitute the human vagina wall (fibroblasts, epithelial cells, and smooth muscle cells). To this end, we tested 41 protocols for their efficacy and reproducibility to retrieve human vaginal cells (Figure 1). We focused on detailed descriptions of the isolation protocols to enable future experiments without missing information. To this end, we analyzed the cell population purity, duplicate consistency, speed (time to first passage at 100% confluency), and the cell viability (number of days per passage). This report may improve the outcome and quality of life for patients in need of vaginal reconstruction by aiding future development of cell-based neovaginal constructs. Furthermore, the consistent and reproducible isolation of cells may further benefit our previously designed sterile and vascularized, human DVM. Lastly, autologous vagina cells carry great promise for the treatment of medical conditions such as Mayer-Rokitansky-Küster-Hauser syndrome or vaginal prolapse, which require partial vaginal reconstruction or support of the vaginal wall.

## 2. Materials and Methods

### 2.1. Approach: Inclusion and Exclusion Criteria

We cross-examined published isolation protocols (Table 1) for common numerators. As an inclusion criterion, the use of enzymatic digestion was required. For additional stimulation of cell type-specific growth and tissue retrieval, other alternatives were considered. Exclusion criteria included the requirement of (1) specialized equipment (i.e., a fluorescence-activated cell sorting system [36] due to bio-incompatibility of fluorescent markers), (2) surgical separation of tissue layers [19,26,39], or (3) missing essential details to reproduce the protocol [38]. Thereby, 41 isolation protocols were included, with 13 protocols for VECs, 24 protocols for SMCs, and 4 protocols for FBs.

VEC isolation was tested for a range of collagenase-I, collagenase-IV, and dispase-II concentrations [34,40,42]. SMC isolation was examined for a range of collagenase-IV concentrations [34,37], and SMC stimulation was attempted using alternative culturing under mechanical forces [44] or hypoxic conditions [45]. Isolation of human vaginal FBs was studied for a range of collagenase-IA concentrations [41] using an alternative direct culturing approach. In addition, increased cell specificity was tested for VECs and SMCs by only dicing the epidermal layer, or smooth muscle and lamina propria, respectively.

### 2.2. Retrieval of Human Vagina Wall Tissue

Vaginal tissue was retrieved from 6 patients during robotic-assisted laparoscopic colpectomy at Amsterdam UMC in Amsterdam between March–August 2024 (Table S1), in accordance with the previous publication [46]. Vaginal tissue was immediately stored in phosphate buffered saline (PBS) pH 7.4 on ice and transported into the laboratory. 

### 2.3. Protocols for Isolation of VECs (By Enzymatic Digestion)

Tissue was aseptically processed in a biological safety cabinet at a biosafety level 2 facility.

#### 2.3.1. VEC Isolation by Enzymatic Digestion

Approximately 1500 mm^3^ of tissue was dissected into fragments of 1–2 mm^3^. Protocols were performed in duplicates. Diced tissue was incubated with 12 mL of collagenase-I (Merck, St. Louis, MO, USA) in a 15 mL tube (Table 2). Enzymatic digestion was performed with:

- 1 mg/mL collagenase-I with/without 2 or 4 mg/mL dispase-II (Merck, St. Louis, MO, USA)
- 1.5 mg/mL collagenase-I with 4 mg/mL dispase-II
- 2 mg/mL collagenase-I with 1, 2 or 4 mg/mL dispase-II

Digestion was performed for 60 min at 37 °C in PBS (Fresenius Kabi, Zeist, The Netherlands), with horizontal placement at the bottom of a water bath while shaking at 80 RPM. The tube was disinfected with ethanol. Digested tissue was filtered through a 70 μm cell strainer and centrifuged for 2 min with 200 RCF at RT. The pellet, supernatant, and recovered tissue were separately dissolved in Dulbecco’s modified Eagle medium (DMEM—Fisher Scientific, Landsmeer, The Netherlands) supplemented with 1% pen/strep (PS—100 U/mL penicillin and 100 μg/mL streptomycin; Life Technologies Europe BV, Bleiswijk, The Netherlands). Cells were incubated in a humidified atmosphere of 95% O_2_ and 5% CO_2_ at 37 °C.

#### 2.3.2. VEC Isolation by Enzymatic Digestion of Diced Epidermal Layer

For VEC isolation, increased cell specificity was tested with diced tissue from the surficial epidermal layer (Table 2; Supplement S2). Roughly 1000 mm^3^ of tissue was digested in a 15 mL tube by 12 mL of the following enzymes:

- 1.5 mg/mL collagenase-I with/without 2 mg/mL dispase-II
- 1 mg/mL collagenase-I with 0.1% collagenase-IV
- 4 mg/mL dispase-II
- 2 mg/mL dispase-II with 0.25% collagenase-IV
- 0.5% collagenase-IV

A 60 min. digestion was performed, and the pellet, supernatant, and tissue were separately cultured.

### 2.4. Protocols for Isolation of Vaginal SMCs

#### 2.4.1. SMC Isolation by Enzymatic Digestion

Roughly 1500 mm^3^ of tissue was dissected into 1–2 mm^3^ fragments. Protocols were performed in duplicates. Diced tissue was digested overnight by 12 mL of collagenase-IV in a 15 mL tube at 37 °C while gently shaking (Table 3). Digestion was performed with the following enzymes:

- 0.1% collagenase-IV
- 0.2% collagenase-IV
- 0.5% collagenase-IV
- 2 mg/mL collagenase-IV

The next day, digested fragments were washed with PBS and centrifuged for 2 min with 400 RCF at RT. The pellet and supernatant were separately dissolved in DMEM or DMEM + Ham-F12 (1:1) (Gibco, Fisher Scientific, Landsmeer, The Netherlands), supplemented with 10% fetal bovine serum (FBS—Gibco, Fisher Scientific, Landsmeer, The Netherlands), 1% PS, and 1% amphotericin-B (2.5 μg/mL, Bio-Reagent, Sigma-Aldrich, St. Louis, MO, USA). Cells were incubated in a humidified atmosphere of 95% O_2_ and 5% CO_2_ at 37 °C.

#### 2.4.2. SMC Isolation by Enzymatic Digestion of Diced Smooth Muscle or Lamina Propria Layer

For SMC isolation, increased cell specificity was tested with diced tissue from smooth muscle (adjacent to the burned adventitia) and lamina propria (Table 3, Supplement S2). Tissue was digested with 0.1% collagenase-IV and cultured at 37 °C and 32 °C in (1) DMEM, (2) in DMEM + Ham-F12 (1:1), or (3) in a specific SMC medium containing DMEM + Ham-F12 (1:1), 5% FBS, 1% PS, 0.05% insulin from bovine pancreas (Sigma, St. Louis, MO, USA), 0.001% epidermal growth factor (EGF; Peprotech, Fisher Scientific, Landsmeer, The Netherlands), and 0.004% human basic fibroblast growth factor (bFGF; Peprotech, Fisher Scientific, Landsmeer, The Netherlands).

#### 2.4.3. SMC Isolation by Cell Attachment Under Agitation or at Low Oxygen Levels

SMC isolation by 0.1% collagenase-IV was alternatively performed under constant agitation at 5 or 150 RPM shaking, as the exertion of mechanical forces can stimulate SMCs. Similarly, isolation by 0.1% or 2 mg/mL collagenase-IV (Table 4) was performed under hypoxic conditions of 2% O_2_ at 37 °C, as this can stimulate SMCs.

#### 2.4.4. SMC Isolation by Alternative Cell Passage

Standard cell passage involved cell detachment by 5 min incubation in trypsin (Gibco, Fisher Scientific, Landsmeer, The Netherlands). However, the strength of cell adhesions varies across cell types. Fibroblasts detach within 0.5–1 min of trypsinization, while most other cells require 3–5 min [47]. Therefore, alternative passaging (Table 5) was tested for the following processes:

- 1 min trypsinization
- 3 min trypsinization
- 5 min trypsinization
- 5 min incubation accutase (Stemcell technologies) with 24 h cell attachment

Trypsinization is normally followed by 24 h of cell adherence before the first medium refreshment. Alternative cell attachment was tested by sequential replating, performing initial cell attachment for 2 h, after which unattached cells were transferred to a second culturing well. After another hour, unattached cells from well two were transferred to a third well and received fresh medium after 24 h. Resulting in cell adherence for 2 h, 3 h, and 24 h.

### 2.5. Protocols for Isolation of Vaginal FBs

#### 2.5.1. FB Isolation by Enzymatic Digestion

Roughly 400 mg of tissue was diced in fragments smaller than 1 mm^3^ (Table 6). Protocols were performed in duplicates. Tissue was washed twice in PBS supplemented with 1% PS and centrifuged for 5 min with 200 RCF at RT. The pellet was collected in a 15 mL tube, and 10 mL of prewarmed pre-mix B was added consisting of 1 mg/mL collagenase-IA (Merck), 5 mg/mL fatty-free acid Bovin Serum Albumin (Merck, Fisher Scientific, Landsmeer, The Netherlands), and 1% PS in DMEM. The diluted pellet was filtered through a 0.22 μm cell strainer, followed by 40 min of digestion at 37 °C, with horizontal placement at the bottom of a water bath and shaking at 80 RPM. The tube was sprayed with 70% ethanol and the digestion mix was added to DMEM supplemented with 10% FBS, 2% PS, and 2% amphotericin-B. The mixture was centrifuged for 10 min with 200 RCF at RT. The pellet and supernatant were separately cultured in DMEM, or in DMEM + Ham-F12 (1:1), supplemented with 10% FBS and 1% PS, and incubated in a humidified atmosphere of 95% O_2_ and 5% CO_2_ at 37 °C.

#### 2.5.2. FB Isolation by Direct Tissue Culturing

FB isolation was alternatively tested by direct culturing of tissue fragments. For isolation of fibroblasts from umbilical cord, an optimized internal protocol involves direct plating of small umbilical cord sections. Fibroblast outgrowth from plated sections typically results in isolated fibroblasts. Therefore, direct plating was tested for small vagina wall sections, and for supernatant after PBS washes without enzymatic digestion. Tissue blocks of 1–2 mm^3^ were dried for 30 min before the addition of DMEM or DMEM + Ham-F12 (1:1). Direct plating was also performed for tissue blocks from the cell strainer.

### 2.6. Immunofluorescent Microscopy

#### 2.6.1. Detection of Fluorescent Vimentin, Desmin and UEA-1

For immunofluorescence microscopy, 0.3 mL of FBs, SMCs, and VECs (P0-P8) were cultured at a density of 1 × 10^5^ cells/mL on Nunc Lab-Tek 8-well Chamber Slides (Fisher Scientific, Landsmeer, The Netherlands) for two days. Cells were fixed for 15 min with Roti Histofix 4% paraformaldehyde (Carl Roth, Karlsruhe, Germany). Slides were washed 2 x 5 min in PBS and stored in 70% ethanol at 4 °C. A heat-induced antigen retrieval step was performed for 10 min in 10 mM Tris (Roche, Woerden, The Netherlands) + 1 mM EDTA (Bio-rad, Lunteren, The Netherlands) at pH = 9.0. Slides were washed 3 x 5 min in PBS while slowly shaking. Aspecific binding was blocked by 10 min incubation with Superblock (ThermoFischer Scientific, Landsmeer, The Netherlands) in a humid slide box at RT. A short and gentle PBS rinse was performed. Next, the slides were incubated in Bright Diluent (VWR, Amsterdam, The Netherlands) with Ulex Europaeus Agglutinin I (UEA-1—VectorLabs, 1:225 concentration, overnight incubation at 4 °C) to identify vaginal endothelial cells, vimentin (Atlas Antibodies, rabbit pAb, 1:1600 concentration, overnight incubation at 4 °C) for FBs, and desmin (Sigma-Aldrich, mouse mAb, 1:330 concentration, overnight incubation at 4 °C) for SMCs. After 30 min of accumulation at RT, the slides were washed 3 x 10 min in PBS while slowly shaking. The slides were incubated for 5 min with 300 ng/mL DAPI in PBS. After a 5 min PBS wash, the slides were embedded with Prolong Gold Antifade Reagent (Life Technologies Europe BV, Bleiswijk, The Netherlands), hardened 1 h at RT, and placed at 4 °C for a couple of hours before analysis with an Olympus BX 41 (Olympus, Tokyo, Japan) light microscope.

#### 2.6.2. Detection of Fluorescent Cytokeratin

Cells were seeded on Nunc Lab-Tek 8-well Chamber Slides, fixed, and stained according to the description for vimentin, desmin, and UEA-1. The slides were incubated with cytokeratin-19 (ThermoFischer Scientific, mouse mAb, 1:20 concentration, overnight incubation at 4 °C) to identify VECs, and counterstained with DAPI. After a 5 min PBS wash, the slides were embedded with Prolong Gold Antifade Reagent, hardened 1 h at RT, and placed at 4 °C for a few hours before analysis with an Olympus BX 41 light microscope.

### 2.7. Cell Morphology by Phase Contrast Microscopy

During isolation, the cell morphology was routinely evaluated using phase contrast microscopy (Olympus, Tokyo, Japan). Morphological assessments were recorded and used as an initial indicator for successful isolation, cell viability, and identification of cell types.

## 3. Results

For the examination of isolation protocols, vaginal wall tissue was obtained from six healthy transgender individuals (Supplement S2). This redundant tissue was collected during a robotic-assisted laparoscopic colpectomy, which was part of their desired medical transition. No modifications to the planned medical procedure were required, and the donation of tissue had no advantageous or adverse effects on the current or future treatments of the patient. No anomalies were identified in the biopsies of the donated vaginal walls by the pathology department. Atrophy was detected in one 24-year-old patient.

### 3.1. Protocol for Isolation of VECs

#### 3.1.1. VEC Isolation by Enzymatic Digestion

**Figure 2:**
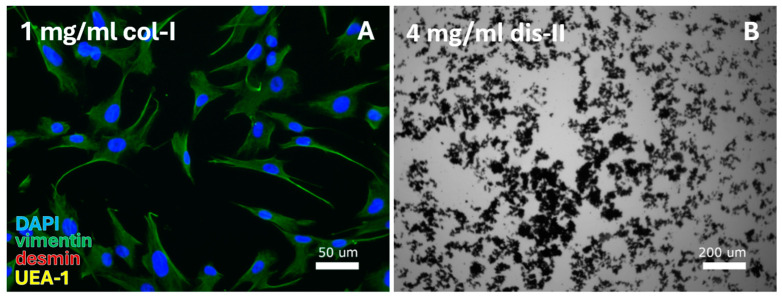
VEC-specific protocols were tested for digestion by a range of collagenase-I (col-I), collagenase-IV, and dispase-II (dis-II) concentrations. This resulted in (**A**) isolation of fibroblasts from the cell pellet after digestion with 1 mg/mL collagenase-I and (**B**) cell debris for use of high (2 or 4 mg/mL) dispase-II concentrations or combined use of dispase-II, collagenase-I, and collagenase-IV. DAPI = cell nuclei (blue), vimentin = fibroblasts (green), desmin = smooth muscle cells, and UEA-1 = endothelial cells (yellow).

VEC isolation from human vaginal wall tissue was examined for seven protocols that relied on the enzymatic action of collagenase-I, dispase-II, and collagenase-IV. In total, one out of seven protocols resulted in the isolation of cells (Table 2A–green blocks), which involved digestion with 1 mg/mL collagenase-I in the absence of dispase (Figure 2A, Table 7A). All cells contained a large, elongated, and spindle-shaped morphology typical of FBs, and were positive for the FB-specific vimentin staining. Cells were first passaged (at 90–100% confluency) after 48 days, and cultures were continued until day 81 at passage 6. Application of higher enzyme concentrations or combined enzyme use resulted in formation of cell aggregates. Aggregates were unable to adhere to the culture plate (Figure 2B), and no cells could be isolated.

Digestion with 1.5 mg/mL collagenase-I and 4 mg/mL dispase-II did not result in isolation. Digestion with 2 mg/mL collagenase-I and 1, 2, or 4 mg/mL dispase-II did not result in isolation.

#### 3.1.2. VEC Isolation by Enzymatic Digestion on Diced Epithelial Layer

Increased cell specificity using six alternative protocols (Table 2B) on diced epithelial layer sections did not result in cell populations.

### 3.2. Protocols for Isolation of Vaginal SMCs

#### 3.2.1. SMC Isolation by Enzymatic Digestion

**Figure 3:**
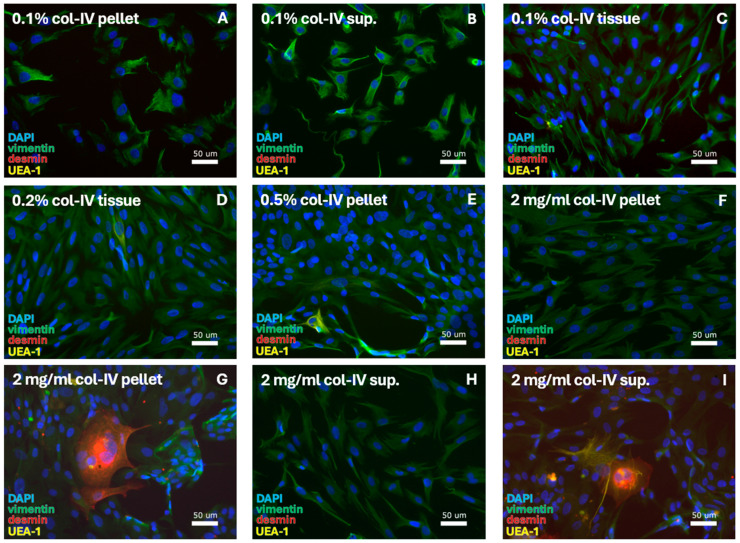
SMC-specific protocols were tested for digestion using a range of collagenase-IV (col-IV) concentrations. This resulted in isolation of FBs. With 0.1% col-IV and culturing in DMEM, FBs were retrieved from the (**A**) pellet, (**B**) supernatant (sup.), and (**C**) tissue. FBs were also retrieved through culturing in DMEM/F12 after isolation with (**D**) 0.2% col-IV from the tissue and (**E**) 0.5% collagenase-IV from the pellet. Digestion with 2 mg/mL col-IV resulted in FBs from (**F**) the pellet, with (**G**) the sporadic detection of muscle cells at quantities <5%, and from (**H**) the supernatant, with (**I**) the sporadic detection of muscle and epithelial cells at quantities <5%. DAPI = cell nuclei (blue), vimentin = fibroblasts (green), desmin = smooth muscle cells, and UEA-1 = endothelial cells (yellow).

SMC isolation was examined for eight protocols that relied on 0.1%, 0.2%, 0.5%, or 2 mg/mL collagenase-IV and were cultured in DMEM (Table 3A) or DMEM/F12 (Table 3B). In total, four out of eight protocols resulted in the isolation of cells (Table 3A,B—green blocks):

- 0.1% collagenase-IV: **isolation in DMEM from pellet, supernatant, and tissue.** With 100% cell purity (Figure 3A–C), cells were identified as FBs from their morphology and positive vimentin staining. Cells were first passaged after 56 days and cultured for 64–81 days until passages 3–12 (Table 7B).
- 0.2% collagenase-IV: **isolation in DMEM/F12 from tissue.**
- With 100% cell purity (Figure 3D) cells were identified as FBs from their morphology and positive vimentin staining.
- 0.5% collagenase-IV: **isolation in DMEM/F12 from pellet.**
- With 100% cell purity (Figure 3E) cells were identified as FBs from their morphology and positive vimentin staining.
- 2 mg/mL collagenase-IV: **isolation in DMEM/F12 from pellet and supernatant.**
- A protocol inconsistency was seen (Figure 3F–I); SMCs or VECs were sporadically observed (less than 5%).

For isolation in DMEM/F12, cell passage was first performed after 21–56 days, and cultures were continued until days 31–95 at passages 3–6 (see Table 7B for specifications).

#### 3.2.2. SMC Isolation by Enzymatic Digestion on Diced Smooth Muscle or Lamina Propria

**Figure 4:**
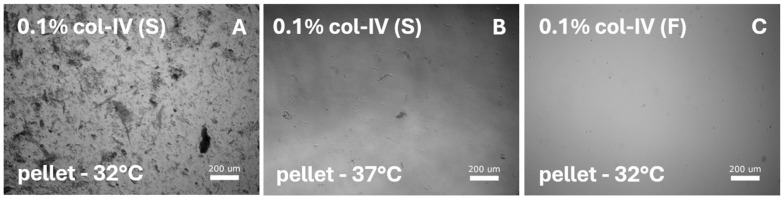
SMC-specific protocols were tested on diced sections from the smooth muscle layer and lamina propria. Digestion with various collagenase-I, collagenase-IV, and dispase-II concentrations was performed and tested for various media and temperatures. All protocols resulted in cell debris. Representative images illustrate digestion with 0.1% collagenase-IV (col-IV) from the cell pellet after culturing in (**A**) SMC-specific medium [S] at 32 °C or (**B**) at 37 °C, and in (**C**) DMEM/F12 medium [F] at 32 °C.

The 12 alternative protocols performed on diced sections from smooth muscle (Table 3C) or lamina propria (Table 3D) with 0.1% collagenase-IV did not result in isolation. However, cell debris was observed (Figure 4).

#### 3.2.3. SMC Isolation by Cell Attachment Under Agitation

Alternative SMC culturing was performed under agitation (Table 4A) by shaking with 5 or 150 RPM after digestion with 0.1% collagenase-IV. Given the absence of cell adherence, this test was terminated after 3 weeks.

#### 3.2.4. SMC Isolation by Cell Attachment at Low Oxygen Levels

**Figure 5:**
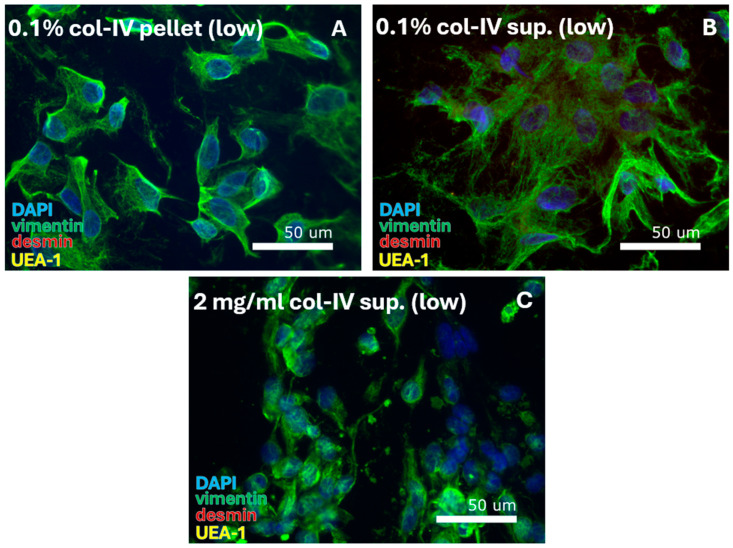
SMC-specific protocols were tested for culturing under hypoxic conditions of 2% oxygen (low). Digestion with 0.1% collagenase-IV resulted in isolation of FBs from (**A**) the pellet and (**B**) the supernatant (sup.). Digestion with 2 mg/mL collagenase-IV resulted in FB isolation from (**C**) the supernatant. DAPI = cell nuclei (blue), vimentin = fibroblasts (green), desmin = smooth muscle cells, and UEA-1 = endothelial cells (yellow).

A second alternative culturing was performed for SMCs under hypoxic conditions at 2% oxygen (Table 4B). Digestion with 0.1% collagenase-IV resulted in isolation from the pellet and supernatant (Figure 5A,B). All cells stained positive for vimentin but showed a non-typical FB morphology that had a folded or curved shape.

Digestion with 2 mg/mL collagenase-IV resulted in isolation from the supernatant (Figure 5C). All cells stained positive for vimentin and had the same atypical curved morphology.

Cells were first passaged after 2–11 days, and cultures were continued for 11–43 days until passages 1–8 (see Table 7B for specifications).

#### 3.2.5. SMC Isolation by Alternative Cell Passage

**Figure 6:**
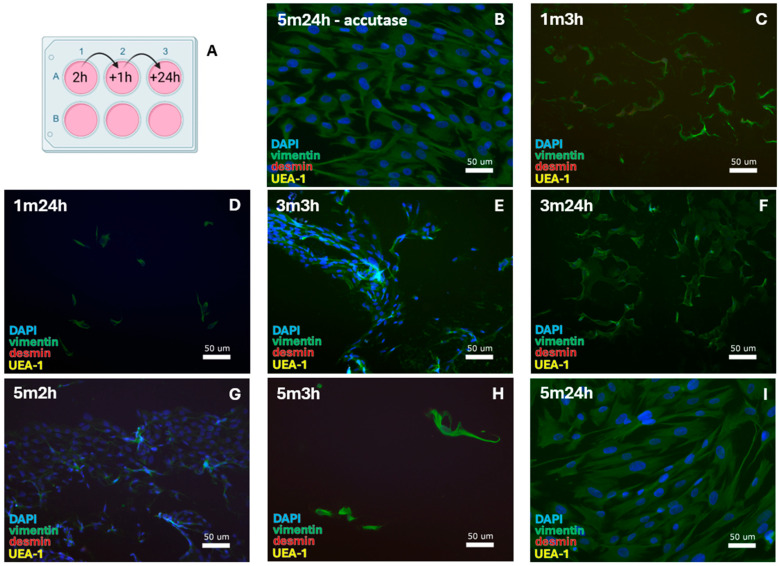
SMC-specific protocols were tested for 0.1% collagenase-IV digestion with (**A**) alternative cell passaging. This resulted in the isolation of FBs after (**B**) 5 min of incubation in accutase and 24 h of cell attachment until the first medium refreshment. After 1 min trypsinization, FBs were isolated with cell attachment for (**C**) 3 h and (**D**) 24 h. For 3 min trypsinization, FBs formed with (**E**) 3 h and (**F**) 24 h cell attachment. For 5 min trypsinization, the same was observed with (**G**) 2 h, (**H**) 3 h, and (**I**) 24 h cell attachment. DAPI = cell nuclei (blue), vimentin = fibroblasts (green), desmin = smooth muscle cells, and UEA-1 = endothelial cells (yellow).

Alternative passaging was tested for isolation of vaginal SMCs, involving 1, 3, or 5 min of trypsinization, and sequential cell attachment (Figure 6A). The results were as follows:

- 1 min trypsinization: isolation by 3 h and 24 h attachment.

After 1 min cell dissociation with 3 h (Figure 6C) or 24 h (Figure 6D) of cell adherence, cells contained a FB morphology typically seen when they are unable to interact with neighboring cells (low cell densities).

- 3 min trypsinization: isolation by 3 h and 24 h attachment.

After 3 min dissociation with 3 h (Figure 6E) and 24 h (Figure 6F) of cell adherence, a higher cell quantity was observed. These cells contained a more typical FB morphology compared to 1 min dissociation.

- 5 min trypsinization: isolation by 2 h, 3 h, and 24 h attachment.

A high cell quantity was retrieved with 2 h (Figure 6G) of cell adherence. These cells showed a typical FB morphology. For 3 h (Figure 6H) or 24 h (Figure 6I) of cell adherence, low quantities of cells were present, displaying the low-density FB morphology.

- 5 min incubation accutase: isolation by 24 h attachment.

Cell populations consistently developed after 24 h of cell attachment (Figure 6B). All cells had a typical FB morphology and stained positive for vimentin. Given the absence of SMCs, the culture was ended on day 4 at passage 0.

All cell populations formed from trypsin-based cell dissociation (Table 5—green blocks) showed protocol consistency and could be identified as FBs by positive vimentin staining. The first cell passage was performed after 2–13 days and the cell culture was continued until days 14–64 at passages 4–12 (see Table 7D for specifications).

### 3.3. Isolation of Vaginal FBs

#### 3.3.1. FB Isolation by Enzymatic Digestion

Isolation of human vaginal FBs was tested for digestion with 2 mg/mL collagenase-IA and culturing in DMEM or DMEM/F12 (Table 6A).

**Figure 7:**
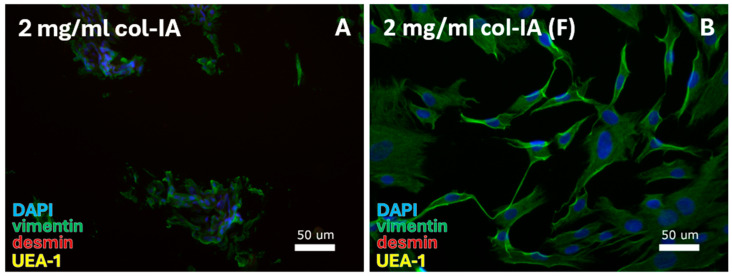
FB-specific protocols were tested for digestion with 2 mg/mL collagenase-IA. FBs were isolated from the cell pellet with culturing in (**A**) DMEM and (**B**) DMEM/F12 (F). DAPI = cell nuclei (blue), vimentin = fibroblasts (green), desmin = smooth muscle cells, and UEA-1 = endothelial cells (yellow).

Both DMEM (Figure 7A) and DMEM/F12 (Figure 7B) resulted in FBs, based on cell morphology and positive vimentin labeling. Cultures were continued until passage 0 after 10 days (Table 7C).

#### 3.3.2. FB Isolation by Direct Tissue Plating

Alternative protocols for FBs involved the direct plating of diced tissue with culturing in DMEM or DMEM/F12 (Table 6B). This did not result in isolation of cells.

## 4. Discussion

Various causes (i.e., Mayer–Rokitansky–Küster–Hauser syndrome, cloacal malformations, gender incongruency, disorder of sex development, cancer, trauma, and genital mutilation) can result in the (partial) absence of a healthy, functioning vagina. The most common medical treatment is the surgical creation of a neovaginal cavity that is lined with a graft. However, these non-vaginal grafts (made from i.e., skin, intestine, or peritoneum) are associated with post-operative complications and complaints due to their insufficient capacity to mimic the physiology of a vagina. Tissue-engineered grafts form a viable alternative, but isolation of host-derived vaginal cells is needed for graft colonization to optimize graft functionality and reduce complications after insufficient implant integration. In vitro, autologous cells have been expanded within 2 weeks from a 1 cm^2^ full-thickness biopsy to 314 cm^2^ of vaginal tissue [48]. Retrieval and in vitro expansion of autologous vaginal cells is an easy and minimally invasive process, and holds great promise for various medical conditions. However, reproducible and consistent protocols are needed to obtain healthy and pure autologous cell cultures. To this end, we tested published isolation protocols with digestion by collagenase and dispase.

Isolation was tested for VECs with collagenase-I, collagenase-IV, and dispase-II [34,40,42], for SMCs with collagenase-IV [34,37], and for FBs with collagenase-IA [41]. We investigated 41 protocols, where two of four FB protocols, one of thirteen VEC protocols, and six of ten SMC protocols resulted in isolation of FBs. No VEC or SMC colonies were isolated, and all cells were classified as FBs of at least 95% purity, based on their spindle-shaped morphology and positive vimentin staining. These unexpected findings will be discussed in depth in the following sections.

### 4.1. VEC Isolation by Enzymatic Digestion

Reports on VEC isolation from human biopsies are scarce. In this study, 13 VEC protocols [34,40] were tested. Digestion with 1 mg/mL collagenase-I resulted in retrieval of cells, which were identified as FBs; thus, VEC isolation was not achieved.

In 2008, epithelial cells were isolated from rabbit vaginal tissue using collagenase-IV and dispase-II [34]. These findings could not be reproduced in our study, which could be explained by the unreported enzyme concentrations, shaking conditions, and centrifugation velocity, or the difference in species. We tested a range of concentrations (0.1–0.5% collagenase-IV, 1–4 mg/mL dispase-II, and 1–2 mg/mL collagenase-I). However, due to unreported conditions, our enzyme concentrations, shaking condition (80 RPM), and centrifugation (2 min with 200 RFT at RT, based on a second report [40] and protocols for other cell types) might be different and thus unsuitable for VEC isolation.

In 2018, epithelial cells were isolated from human vaginal tissue using 1.5 mg/mL collagenase-I and 4 mg/mL dispase-II, and expansion in EpiLife medium [40]. Using the same enzymes and concentrations, we found that combinatorial digestion resulted in small lumps of non-proliferating cell debris. Due to unreported centrifuge settings and our alternative cell medium, this irreproducibility might be related to experimental differences. As VECs are sensitive to microenvironmental changes, our medium might not be suitable. Furthermore, instead of surgical separation of tissue layers by an expert [19,26,39], we tested increased cell specificity by dicing from epidermal tissue. This did not result in isolation of cells from the relatively small quantities of tissue. Lastly, FBs are burdensome to in vitro cultures because they are insensitive and overgrow most other cell types, even with low levels of contamination [49]. Isolation of primary cells is therefore most likely to result in FBs.

Other reports on successful isolation of human VECs from biopsies that were not tested in this study might show reproducible results. For example, two thorough and extensive reports from the Bolduc group have described VEC isolation [19,26]. With the inclusion of 41 protocols and optimization of cell specificity, enzyme concentrations, temperatures, medium composition, non-static or hypoxic culturing, and alternative passaging, their method was excluded due to the use of other digestive enzymes. We want to stress that their use of irradiated fibroblasts as a feeding layer for VECs might form a crucial addition to the avoidance of FB overgrowth.

Furthermore, this study tested VEC isolation with separate or simultaneous use of digestive enzymes. Cells (specifically FBs) were retrieved with 1 mg/mL collagenase-I, but sequential enzyme digestion was not investigated. An additional digestion with low dispase-II or collagenase-IV concentrations could provide a valuable modification. Their sequential use might be required to separate epithelial cells from FBs, thereby offering a promising solution to the current shortage of reproducible protocols for VEC isolation from human biopsies.

### 4.2. SMC Isolation

#### 4.2.1. SMC Isolation by Enzymatic Digestion

For eight tested SMC protocols [37,50], cells isolation was achieved with 0.1%, 0.2%, 0.5%, and 2 mg/mL collagenase-IV. Digestion with 0.1% collagenase-IV resulted in the most viable FBs (7.5 million cells/cm^2^), which were terminated at P12 to prevent phenotypical modifications at higher passages. Isolation of SMCs was not achieved.

In 2002, human SMCs were isolated from corpora cavernosa using mechanical dispersion and 1 mg/mL bacterial collagenase [50]. The irreproducibility of these findings might be explained by the use of a different (penile and vaginal) tissue type, as well as unreported mechanical conditions and collagenase types.

In 2020, human vaginal SMCs were isolated with 2 mg/mL bacterial collagenase-IV [34,37]. We encountered FBs and 5% SMCs with 2 mg/mL collagenase-IV. Combined with sub-selection of SMCs, this protocol might be suitable as protocol inconsistency occasionally resulted in some SMCs. Furthermore, FB overgrowth was observed for increasing passage numbers, indicating that SMC isolation requires a specific medium, boosting of SMC proliferation, or the removal of FB contamination. We tested a SMC-specific medium that contained all the supplements of commercial SMC medium (i.e., smooth muscle growth supplement from Gibo, Smooth Muscle Cell Growth Medium 2 from PromoCell). This SMC-specific medium had no effect on the isolation.

#### 4.2.2. SMC Isolation Under Agitation or at Low Oxygen Levels

Mechanical stretching of cells can guide cell growth and shape, and stimulate SMC proliferation [44]. We tested culturing under constant shaking at 5 or 150 RPM and found no cell attachment, even after three weeks. This might be resolved by initiating shaking after cell adherence. Studies have also demonstrated a hypoxia-mediated effect on SMC proliferation [45]. In our study, culturing at 2% oxygen resulted in isolation of FBs with an atypical morphology. Therein, the absence of SMCs might be explained by hypoxia-mediated effects on the phenotype [51,52] and proliferation of FBs [53]. Phenotypical changes could be evaluated using hypoxia-related markers such as hypoxia inducible factor (HIF) 1α [54]. This could provide insights into whether the hypoxia-stimulated effect on SMC proliferation was insufficient to compensate for the enhanced FB proliferation.

#### 4.2.3. SMC Isolation by Alternative Cell Passage

Traditionally, trypsin is used for cell passaging to dissociate cell–cell adhesions and to detach cells from culture plates. The strength of cell adhesions, and thus the required time for cell detachment and adherence, vary across cell types. FBs detach within 0.5–1 min, whereas most cell types require 3–5 min [47]. We tested whether modified cell detachment (1, 3 or 5 min) and adherence (2, 3 or 24 h) could assist in the isolation of SMCs by removing FB contaminants. Isolation of SMCs was not achieved, but alternative culturing was initiated at passage 4, at which point FBs might have already overgrown SMCs. Furthermore, FBs were removed from 2 h cell attachment cultures after 1 and 3 min of trypsinization, indicating that consistent short trypsinization (0.5–1 min) and adherence (15–20 min according to ResearchGate forums) may aid in FB removal.

#### 4.2.4. SMC Isolation by Digestion of Smooth Muscle or Lamina Propria

Vaginal SMCs are primarily found in smooth muscle and lamina propria [55,56]. Instead of surgical separation of tissue layers, we tested increased specificity by dicing from these layers. In addition, stimulation of SMC proliferation was attempted with various media and alternative cultivation at 32 °C, as low temperatures can prevent FB contamination [57]. No cells were retrieved, likely due to the insufficient tissue volume of 25 μL (1 × 10^6^ cells) for overnight enzymatic digestion. This could be resolved with larger tissue volumes.

### 4.3. VEC or SMC Isolation by Marker-Based Techniques

Additional cell isolation techniques are available that use specific fluorescent or magnetic markers for sorting. Fluorescence-activated cell sorting (FACS) requires the binding of fluorescent antibodies to specific cell markers. Labeled cells can then be detected and isolated in a flow cytometry device, using an electrical charge and an electromagnetic field for the charge-based sorting of cells. FACS-based isolation of VECs and SMCs was considered for this study, as it enables high-purity sorting of rare cells in mixed cultures [58]. However, FACS can cause apoptosis and adversely affect cell viability for subsequent experiments. FACS could also cause bio-incompatibility issues in clinical translation, when fluorescently labeled autologous cells would be used for graft seeding with implantation in humans. This could result in lethal complications.

Magnetic-activated cell sorting (MACS) offers an alternative with magnetic particles that bind to specific cell markers. A magnetic field sorts the targeted magnetic cells. Once separated, magnetic beads can be removed from cells, thereby preventing the potential bio-incompatibility risks of FACS. However, MACS requires specialized equipment and can rupture cell membranes, causing irreversible damage. Thus, this study focused on the use of accessible and biocompatible digestive enzymes.

### 4.4. FB Isolation by Enzymatic Digestion

The tested protocols for isolation of human FBs [41] resulted in FB cultures. No cultures formed from explants of direct plating [27], likely due to detrimental collagenase-IA effects that are otherwise arrested by PBS washing steps.

VEC and SMC protocols showed that enzyme concentrations have a minimal effect on FB cultures. All isolated cultures were identified as FBs, and SMCs were only observed in FB cultures after digestion with high collagenase-IV concentrations. Because FBs have great resilience, their viability and resulting ease of isolation are minimally impacted by factors such as collagenase concentration. A total of nine efficient and consistent protocols were found for isolation of human vaginal FBs from cell pellets, supernatant, or tissue under 13 different conditions.

### 4.5. Quality of Donated Vagina Wall Tissue

The isolation protocols were tested on human vaginal wall tissue from six healthy transgender donors. The donors were relatively young (20–33 years old) and six were included in order to have replicates for biological differences between individuals. The retrieved vaginal tissue was considered healthy as no pathological anomalies were found. However, atrophy was identified in one vaginal biopsy. Vaginal atrophy has been reported in 50% of genotypical women above 40 years of age (menopausal age) and is thus relatively common [59]. Donated tissue for experimental use is typically collected from deceased patients or after surgery, thereby limiting vaginal wall donors to adults. Among 18+ genotypical women, approximately half are beyond menopausal age [60]. Statistically, over 25% of donated vaginal tissue would thus contain signs of atrophy. This also means that for clinical vaginal reconstruction, autologous cells would be retrieved for 25% of patients from atrophic tissue. Therefore, our donors are considered representative. Epithelial atrophy has been reported as a side-effect of (long-term) presurgical androgen exposure [61], which explains its occurrence in a young patient. Hormone replacement therapy has also been associated with prostatic metaplasia (formation of glandular structures in the epithelium or subepithelial stroma) [61,62], but this was not observed in our samples. Medical history was therefore considered not to impact cell isolation, but young age might have been beneficial.

### 4.6. Impact

Vaginoplasty is currently considered the ideal solution for treating vaginal disorders and medical conditions, but it is accompanied by many limitations and drawbacks. Tissue-engineered solutions carry great potential as alternative, but their success relies on the use of autologous cells and their efficient and reliable isolation. These protocols are scarce for retrieval from human vaginal tissue. We found 13 irreproducible VEC protocols that likely require the addition of sequential enzymatic digestion, mechanical separation of tissue layers, VEC-specific medium, and a feeding layer to prevent FB overgrowth. The 10 irreproducible SMC protocols likely require the addition of mechanical separation of tissue layers and other SMC-specific medium. We found nine efficient and reliable protocols for FBs, of which 0.1% collagenase-IV demonstrated the best result. All successful protocols are clinically attractive in terms of efficiency, large-scale expansion, cell source availability (for cell bank establishment), and potential uses in vaginal transplantation without the risk of immunorejection.

Currently, several (commercial) tissue-engineered products with autologous FBs have been tested [15,40,41,63] and applied in clinical studies [18,64,65]. Moreover, the safety and long-term use of cell-based therapy has been widely shown. The FB cultures that we isolated were capable of active growth and efficient expansion, which are important for in vitro-cultured vaginal transplants. Furthermore, we previously demonstrated the creation of a human vaginal wall-derived matrix through chemical decellularization [56]. These matrices carry high clinical potential considering (1) their acellular properties, which allow transplantation without the risk of immunorejection, (2) their natural characteristics in terms of proteins, morphology, and most biomechanical properties, and (3) their mucus-producing capacity. The isolation of autologous vaginal FBs represents a first step toward the clinical translation of these human vaginal wall-derived scaffolds, serving as an example of many potential solutions to fulfill unmet medical needs in terms of neovaginal reconstruction. However, the effective functionalization of these scaffolds likely depends on autologous SMCs for adequate biomechanical properties.

The irreproducible isolation of VECs and SMCs from human vaginal biopsies remains a limitation. Functionalized biomaterials for vaginal reconstruction with autologous cell seeding are limited to a handful of studies. These studies have reported seeding with FBs [18], VECs [48], or VECs and SMCs [34,55]. However, these biomaterials were only studied once, and biomaterial seeding was not compared for different cell types (except in co-culture). Therefore, the role of autologous VECs, SMCs, and FBs in the functionality of a tissue-engineered graft, and its chance of in vivo success, remains to be investigated. To investigate these roles, the future development of reproducible isolation protocols is of great importance. The success of FB isolation by many protocols and the FB overgrowth encountered in our study suggest that the difficulty in VEC and SMC isolation lies not in retrieving the cells from tissue but in separating them from FB contamination.

It can be hypothesized that SMCs may play an essential role, as they impact the vaginal wall’s elasticity: a biomechanical property that requires close mimicking in tissue-engineered solutions to provide an adequate vaginal replacement for childbirth and penetrative sex. Lastly, the isolation of autologous cells for prevascularization and innervation are additional considerations for vaginal reconstruction.

## 5. Conclusions

For the creation of a neovagina, vaginoplasty is considered the most ideal solution. However, this intervention is associated with considerable complications caused by unsuitable physiological properties in grafts that are typically made from skin, intestine, or peritoneum. Various tissue-engineered alternatives have become available, but cell seeding is desired to prevent complications upon their implantation. With this comprehensive comparative study, we derived nine efficient and reproducible protocols for isolation of FBs and found that 0.1% collagenase-IV provides a reliable source of autologous FBs. We also identified inconsistent and unreproducible methods for isolation of VECs and SMCs, and identified solutions to prevent FB overgrowth. These findings aid the clinical translation of cell-based, tissue-engineered solutions to fulfill unmet medical needs in vaginal reconstruction and support. Nevertheless, future development of reproducible isolation protocols for VECs and SMCs might be crucial to achieve adequate matrix functionalization and thus increase the chance of successful clinical implementation.

## Figures and Tables

**Table 1:** Assessment of published isolation protocols on enzymatic digestion (concentration and time), cell retrieval (method of biopsy/tissue sampling; mechanical digestion before enzymatic digestion), replicate consistency (outcome tested for a number of replicates), and testing of cell viability. √ = reported; - = unreported.

Study	Cell Type (s)	Digestion		Cell Retrieval	Consistency	Viability
		conc.	time			
**De Philippo** [34]	SMC/VEC	-	-	-	-	√
**Jakubowska** [26]	FB/VEC	√	√	-	-	-
**Li** [36]	FB/SMC/VEC	√	√	√	-	√
**Maseroli** [37]	SMC	√	√	√	-	√
**Nodale** [38]	FB	-	-	-	-	√
**Orabi** [19]	VEC	√	√	-	-	-
**Ruiz-Zapata** [39]	FB	√	√	-	-	-
**Sartoneva** [40]	VEC	√	√	-	-	√
**Skala** [41]	FB	-	√	√	-	√
**Yuan** [42]	VEC	√	√	-	-	√
**Zhu** [43]	VEC	-	-	-	-	√

**Table 2:** VEC isolation protocols were tested for digestion by collagen-I, dispase-II, and collagenase-IV. (**A**) Overview of the examined enzyme concentrations for full thickness vagina tissue. (**B**) Isolation protocols performed on diced sections from epithelial layers to increase specificity.

**A**	**Layer (s)**	**Collagenase-I (mg/mL)**	**Dispase-II (mg/mL)**	**Collagenase-IV (%)**
**1**	Full thickness	1	-	-
**2**		1	2	-
**3**		1	4	-
**4**		1.5	4	-
**5**		2	1	-
**6**		2	2	-
**7**		2	4	-
**B**	**Layer (s)**	**Collagenase-I (mg/mL)**	**Dispase-II (mg/mL)**	**Collagenase-IV (%)**
**S1**	Epidermal layer	1.5	-	-
**S2**		1.5	2	-
**S3**		1	-	0.1
**S4**		-	4	-
**S5**		-	2	0.25
**S6**		-	-	0.5

**Table 3:** SMC protocols were tested with collagenase-IV. Overview of the enzyme concentrations with culturing in (**A**) DMEM and (**B**) DMEM/F12. Increased cell specificity was tested on diced tissue from (**C**) smooth muscle and (**D**) lamina propria.

**A**	**Medium**	**Collagenase-IV (%)**	**Temperature (** **°** **C)**
**1**	DMEM	0.1	37
**2**		0.2	37
**3**		0.5	37
**4**		2 mg/mL	37
**B**	**Medium**	**Collagenase-IV (%)**	**Temperature (** **°** **C)**
**F1**	DMEM/F12	0.1	37
**F2**		0.2	37
**F3**		0.5	37
**F4**		2 mg/mL	37
**C**	**Medium**	**Collagenase-IV (%)**	**Temperature (** **°** **C)**
**SM1**	DMEM	0.1	37
**SM2**	DMEM/F12		
**SM3**	SMC-specific		
**SM4**	DMEM		32
**SM5**	DMEM/F12		
**SM6**	SMC-specific		
**D**	**Medium**	**Collagenase-IV (%)**	**Temperature (** **°** **C)**
**LP1**	DMEM	0.1	37
**LP2**	DMEM/F12		
**LP3**	SMC-specific		
**LP4**	DMEM		32
**LP5**	DMEM/F12		
**LP6**	SMC-specific		

**Table 4:** Alternative SMC protocols were performed under modified culturing conditions. Overview of the applied modifications, involving (**A**) digestion with 0.1% collagenase-IV followed by shaking at 5 or 150 RPM, or (**B**) digestion with 0.1% or 2 mg/mL collagenase-IV followed by culturing at 2% oxygen levels.

**A**	**Layer (s)**	**Alteration**	**Collagenase-IV (%)**
**Full thickness**		5 RPM shaking	0.1
		150 RPM shaking	
**B**	**Layer (s)**	**Alteration**	**Collagenase-IV (%)**
**Full thickness**		2% oxygen	0.1
			2 mg/mL

**Table 5:** SMC isolation protocols were performed with alternative passaging. This involved (**A**) 1 min trypsinization, (**B**) 3 min trypsinization, or (**C**) 5 min trypsinization. Each condition was tested for 2 h, 3 h, and 24 h of cell adherence before a first medium change was performed.

**A**	**Layer(s)**	**Detachment (min)**	**Adherence (h)**
**1m2h**	Full thickness	1	2 h
**1m3h**			3 h
**1m24h**			24 h
**B**	**Layer(s)**	**Detachment (min)**	**Adherence (h)**
**3m2h**	Full thickness	3	2 h
**3m3h**			3 h
**3m24h**			24 h
**C**	**Layer(s)**	**Detachment (min)**	**Adherence (h)**
**5m2h**	Full thickness	5	2 h
**5m3h**			3 h
**5m24h**			24 h

**Table 6:** FB protocols were tested for (**A**) digestion with 1 mg/mL collagenase-IA or (**B**) by direct culturing of tissue. Culturing was performed in DMEM and in DMEM/F12.

**A**	**Layer(s)**	**Medium**	**Collagenase-IA (mg/mL)**
**1**	Full thickness	DMEM	**1**
**2**		DMEM/F12	
**B**	**Layer(s)**	**Medium**	**Collagenase-IA (mg/mL)**
**P1**	Full thickness	DMEM	**-**
**P2**		DMEM/F12	

**Table 7:** Overview of all retrieved cell populations. (**A**) VEC protocols: cells were retrieved by digestion with 1 mg/mL collagen-I from cell pellet. (**B**) SMC protocols: cells were retrieved by digestion with 0.1%, 0.2%, 0.5%, and 2 mg/mL collagen-IV. (**C**) FB protocols: cell retrieval by 2 mg/mL collagenase-IV for culturing in DMEM and DMEM/F12. (**D**) Alternative cell passage tested for SMCs.

**A**	**Retrieval**	**Figure**	**Collagenase I Concentration**	**Modification**	**Consistent**	**Morphology**		**Purity**	**Passage 1 (days)**	**Cell Viability (days/passage)**	
pellet		2A	1 mg/mL	-	yes	Typical FB		100%	48	P6	13.5
**B**	**Retrieval**	**Figure**	**Collagenase IV Concentration**	**Modification**	**Consistent**	**Morphology**		**Purity**	**Passage 1 (days)**	**Cell Viability (days/passage)**	
pellet		3A	0.1%	-	yes	Typical FB		100%	56	P12	5.3
supernatant		3B								P3	27
tissue		3C								P7	13
tissue		3D	0.2%	-	yes	Typical FB		100%	49	P3	10.3
pellet		3E	0.5%	-	yes	Typical FB		100%	56	P4	23.8
pellet		3F+G	2 mg/mL	-	no	Typical FB		>95%	21	P6	5.8
supernatant		3H+I									
pellet		4A	0.1%	2% oxygen	no	Atypical FB		100%	2	P8	5.4
supernatant		4B									
supernatant		4C	2 mg/mL	2% oxygen	no	Atypical FB		100%	11	P1	11
**C**	**Medium**	**Figure**	**Enzyme Concentration**	**Consistent**		**Morphology**		**Purity**	**Passage 1 (days)**	**Cell Viability (days/passage)**	
DMEM		7A	2 mg/mL Collagenase-IA	no		Typical FB		100%	10	P0	10
DMEM/F12		7B									
**D**	**Detachment**	**Figure**	**Adherence**	**Consistent**		**Morphology**		**Purity**	**Passage 1 (days)**	**Cell Viability (days/passage)**	
1 min		5C	3 h	yes		Low density	Atypical FB	100%	13	P7	5
		5D	24 h						2	P4	4.3
3 min		5E	3 h	yes		Low density	Atypical FB	100%	5	P8	3.6
		5F	24 h			Typical FB			2	P4	3.5
5 min		5G	2 h	yes		Typical FB		100%	9	P6	6.3
		5H	3 h			Low density	Atypical FB		2	P8	4.4
		5I	24 h							P12	5.3

## Data Availability

Data can be shared upon request from the corresponding authors.

## Supplementary Materials

The following supporting information can be downloaded at: https://www.mdpi.com/article/10.3390/cells14020076/s1. Supplement S1: Tissue donor characteristics. Supplement S2: Diced tissue layers for specificity of cell type.
