# Supplementary material for: Reproducibility and Consistency of Isolation Protocols for Fibroblasts, Smooth Muscle Cells, and Epithelial Cells from the Human Vagina
Source: Cells. 2025 Jan 8;14(2):76. doi: 10.3390/cells14020076 (PMC11763730; doi:10.3390/cells14020076)

Supplement 1

Tissue donor characteristics.

Patient	Age (years)	Current Procedure	Pathological findings	Androgen Exposure (years)	Co-morbidity
**VAG017**	24	Total laporoscopic<br>hysterectomy<br>bilateral salpingo-<br>oophorectomy<br>Robot Colpectomy	NA	2.6	GD
**VAG018**	33		NA	1.6	GD
**VAG019**	20		NA	2.2	GD
**VAG021**	24		No vaginal anomalies, atrophy	3.6	GD
**VAG023**	30		NA	1.7	GD
**VAG027**	24		NA	4.4	GD<br>Autism<br>depression

Supplement 2

Diced tissue layers for specificity of cell type.

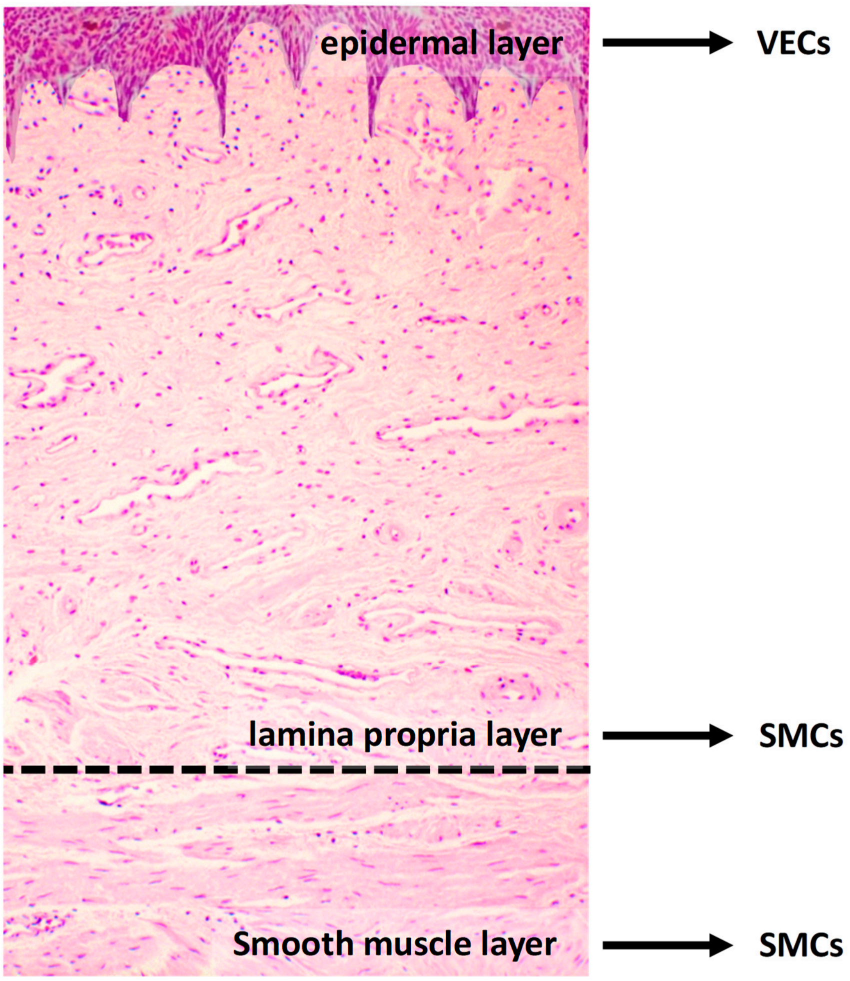

Supplement: Supplementary file 1 [file cells-14-00076-s001.zip › cells-3381786-supplementary.pdf]
